# The association between increased fetal movements in the third trimester and perinatal outcomes; a systematic review and meta-analysis

**DOI:** 10.1186/s12884-024-06547-3

**Published:** 2024-05-15

**Authors:** Sedigheh Hantoushzadeh, Omid Kohandel Gargari, Marzieh Jamali, Fatemeh Farrokh, Nasim Eshraghi, Fatemeh Asadi, Masoumeh Mirzamoradi, Seyed Jafar Razavi, Marjan Ghaemi, Sudabeh Kazemi Aski, Zahra Panhi, Gholam Reza Habibi

**Affiliations:** 1 Vali-E-Asr Reproductive Health Research Center, Family Health Research Institute, Tehran University of Medical Sciences, Tehran, Iran; 2 Student Research Committee, School of Medicine, Alborz University of Medical Sciences, Karaj, Iran; 3 Gene Therapy Research Center, Digestive Diseases Research Institute, Tehran University of Medical Sciences, Tehran, Iran; 4 Department of Gynecology and Obstetrics, Mahdiyeh Hospital, Shahid Beheshti University of Medical Sciences, Tehran, Iran; 5 Reproductive Health Research Center, Department of Obstetricsand Gynecology, School of Medicine, Guilan University of Medical Sciences, Rasht, Iran; 6 Farzan Artificial Intelligence Team, Farzan Clinical Research Institute, Tehran, Iran

**Keywords:** Fetal movement, Fetal demise, Pregnancy, Systematic review

## Abstract

**Background:**

Fetal movement monitoring is one of the strategies used to assess the fetus’s health. Until now, most studies focused on the decreased fetal movement and neonatal outcome, although this systematic review and meta-analysis is designed to assess the association between increased fetal movements (IFM) with perinatal outcomes.

**Method:**

The electronic databases including PubMed, Scopus, Web of Science, and EMBASE were systematically searched for studies investigating the perinatal outcome of women with increased fetal movements from inception to July 2023. Following that, a random-effect meta-analysis model was used to obtain the combined diagnostic and predictive parameters including perinatal mortality (still birth and early neonatal mortality), operative delivery, Apgar score, neonatal resuscitation at birth and NICU Admission.

**Results:**

After the initial screening, seven studies examining the association between increased third trimester fetal movement and various perinatal outcomes were included. Meta-analysis revealed a significant reduction in the risk of cesarean delivery among patients with IFM compared to controls, suggesting a potential protective effect during childbirth. However, no statistically significant difference was observed in birth weight, small or large for gestational age births, neonatal intensive care unit admission, maternal age, umbilical cord around the neck, gestational diabetes mellitus, and hypertension, indicating that IFM may not be a major predictor of adverse perinatal outcomes or maternal conditions. Notably, IFM was significantly associated with a higher likelihood of labor induction.

**Conclusion:**

The findings suggest that IFM may have a protective effect against cesarean delivery. Additionally, IFM does not appear to be significantly associated with maternal age, umbilical cord around the neck, gestational diabetes mellitus and hypertension. However, the observed significant association with labor induction warrants further investigation.

**Supplementary Information:**

The online version contains supplementary material available at 10.1186/s12884-024-06547-3.

## Background

Adverse perinatal outcomes are the main causes of neonatal mortality and morbidity. It is estimated that 2.5 million late gestation stillbirths and 3 million neonatal deaths occur globally each year [1]. Moreover, over 40% of all stillbirths that happen during labor and delivery could have been prevented with improved fetal monitoring and access to emergency obstetric care when needed [2]. Various factors may contribute to stillbirth, including maternal health and obstetric condition, sociodemographic and economic status, congenital abnormalities, pregnancy disorder, and placental dysfunction [3, 4]. It seems crucial to determine the modifiable risk factors to develop prevention strategies and reduce the risk of stillbirth [5].

Fetal well-being can be monitored by the number of fetal movements being perceived by the mother [6]. Reduced fetal movement (RFM) is typically defined as a decrease in the mother’s perception of fetal movements or a change in the pattern of normal movement [7]. A great number of studies that evaluated the relationship between maternal understanding of fetal activity and pregnancy outcomes, were focused RFM. There is some evidence to support that RFM is associated with poor pregnancy outcomes [8]. It was estimated that more than half of the pregnant women who face stillbirth understood the reduction in fetal movements before the diagnosis. The likely mechanism behind RFM was explained as impaired placental function, which led to increased inflammation and limited blood supply in fetuses experiencing RFM [9]. So, guidelines have been developed based on the best recent findings, to provide recommendations for clinicians to manage women with reduced fetal movement [10, 11].

Many studies have been conducted to identify modifiable factors associated with stillbirth, which could lead to direct interventions to reduce the incidence. These studies have examined various factors, encompassing both maternal and fetal aspects, that may contribute to the occurrence of stillbirth. Some of the modifiable factors that have been explored include maternal weight, illicit drug use, the quality of obstetrical care during pregnancy, sleeping conditions, and the condition of being small for gestational age (SGA) [12–14].

Regarding the maternal perception of fetal movements, some clinical data indicated that increased movements are associated with adverse perinatal outcomes [15, 16]. But these data are currently insufficient and sparse [17]. In addition, there is no systematic review that evaluated the risk of adverse outcomes such as stillbirth among women reporting this symptom, and also, it is still unclear how to reduce the risk. The objective of this study is to assess if there is any association between increased fetal movements and perinatal outcomes (included: neonatal mortality, neonatal intensive care unit (NICU) admission, fetal distress, low Apgar score). As a result of this information, medical professionals would be able to provide pregnant women with more comprehensive care, and women would also be able to receive more detailed information to reduce their anxiety.

## Method

### Search strategy

We conducted this systematic review while fully adhering to the guidelines available at the Preferred Reporting Items for Systematic Reviews and Meta-Analyses (PRISMA). To identify the published studies of interest, we prepared a search strategy comprising strings of keywords related to our study’s objectives from inception to July 2023, provided as supplementary file S1. PubMed, SCOPUS, Embase, and Web of Science databases were systematically searched for record identification.

### Selection criteria

To appropriately investigate the identified studies for eligibility, we considered a framework for the investigation of risk of exposure with health outcomes in studies, known as PICO (Population, Intervention, Comparator, Outcomes). We only considered English observational studies (i.e., case-control studies, cohorts, and cross-sectional studies) that investigated perinatal outcomes among women with increased fetal movements (IFM). Therefore, all interventional studies, case series, case reports letters to the editors, meetings, and conference abstracts or proceedings, surveys, editorials, and reviews were excluded.

### IFM is diagnosed using assessment surveys, to avoid missing studies we also included studies recruiting mothers with self-reported IFM

The population in our study were pregnant women with an age > 18 years old, gestational age over 20 weeks, and a singleton, non-anomalous pregnancy. Moreover, pregnant women under age 18, gestational age under 20 weeks, Multiple pregnancies, any established fetal anomalies, and any maternal medical conditions demanding medications that can affect fetal movement were excluded.

Patients should have learned how to measure fetal movements and report them in a specific time interval planned by researchers in each article, or their fetal movements should be measured by questionnaire. Either IFM sensation, surveys and interviews were considered acceptable for IFM assessment. The control groups should be selected from healthy population pregnancies with gestational age-matched (± 7 days) with the intervention group and normal pregnancy outcome. All articles must measure the pregnancy outcomes concerning Stillbirth.

Inclusion criteria:

1. English observation studies (Cohort, cross-sectional and case-control).
2. pregnant women with an age > 18 years old, gestational > 20 weeks,
3. Singleton and non-anomalous pregnancy.
4. Included patients with IFM.
5. Reported perinatal outcomes.

### Data collection and analysis

The study selection, quality assessment, and data extraction were carried out under the supervision of the senior author. We initially collected the identified records from the four mentioned databases and checked for duplicates using the 20th version of the Endnote software package. Then using the duplicate removal tool provided by Rayyan Incorporation10, any remaining duplicate records were manually removed. Next, two authors independently screened the resulting studies based on their titles and abstracts, removing those deemed irrelevant. They screened the records passing through the first round based on their full texts, excluding the ineligible studies. If discrepancies occur in the study selection stages, they are resolved by senior author recommendations.

two authors independently extracted the data from the eligible studies using a pre-specified flexible data extraction form in an Excel Microsoft office spreadsheet. These data included the study’s first author, country, the year it was conducted, study type, method assessment time, fetal movement assessment period estimated outcome, total sample size, case group size, control group size, age case (mean/SD), age control (mean/SD), gestational age range, method of assessment, number of women who experienced increased fetal movements, the study hypothesis and the outcome based on the method of assessment.

Outcomes include type of delivery (cesarean delivery, induced or natural vaginal delivery), birth weight, small for gestational age (SGA), large for gestational age (LGA), neonatal intensive care unit (NICU) admission, maternal age, umbilical cord around the neck (UCNA), gestational diabetes mellitus (GDM), hypertension (HTN), and labor induction.

Adverse perinatal outcomes were defined as perinatal mortality (still birth and early neonatal mortality defined as newborn death within the first 7 days), operative delivery (cesarean section or vacuum) due to fetal distress, Apgar min 5 < 7, neonatal resuscitation at birth (including both invasive ventilation such as mechanical ventilation; non-invasive ventilation such as oxygen therapy, nasal CPAP, high flow), and NICU Admission. In case of any discrepancies, it was resolved by senior third authors. The authors of included articles were contacted to provide additional information.

For outcomes with two or more studies random effect model was used for data pooling and calculating summary estimates. Risk ratio and odds ratio would be used for dichotomous outcomes and the standard mean difference would be used for continuous variables. According to the Cochrane handbook, two studies could be used to perform meta-analysis if their results are sufficiently similar [18]. The quality of the included studies was assessed by utilizing the tools recommended by the Joanna Briggs Institute (JBI) [19, 20]. The quality assessment table is provided in supplementary file S2.

## Results

### Study selection

Database search resulted in 6292 studies and after duplicate removal 3505 studies underwent title and abstract screening. 98 studies were selected for eligibility assessment and full-text screening. Finally, 7 studies were selected to be included in this study. Two studies, although reported fetal movement, did not have a control group and were excluded [21, 22], one study was excluded because it did not report neonatal outcomes [23] (Fig. 1).

### PRISMA 2020 flow diagram for new systematic reviews which included searches of databases and registers only

**Fig. 1:**
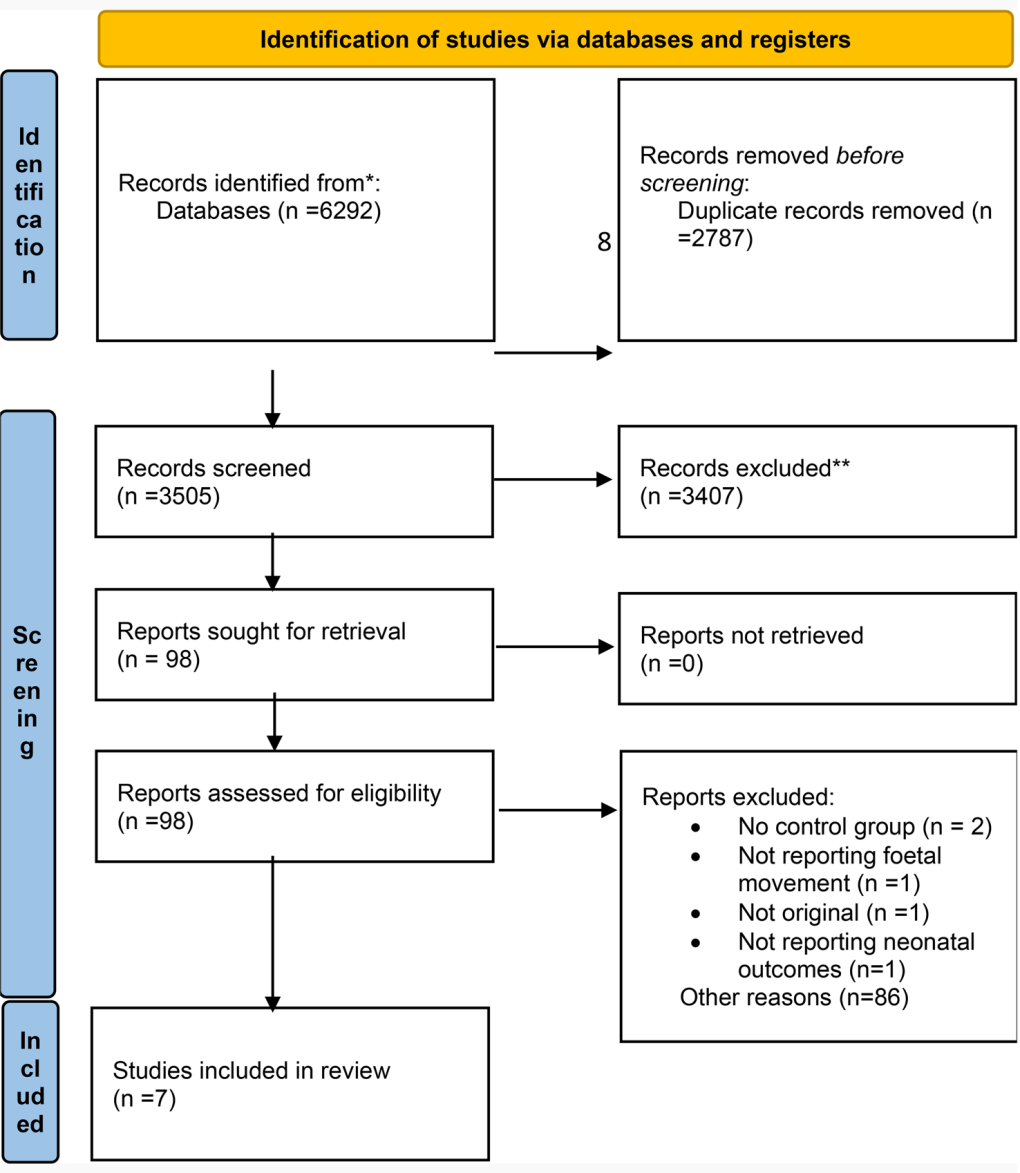
Flow diagram of the recruiting studies according to PRISMA

### Study characteristics

Four out of seven included studies were cohort studies designed to compare patients with increased fetal movement with regular pregnancies [24–27]. Three studies were case-control studies which compared patients with adverse neonatal outcomes with controls and reported increased fetal movement as one of their outcomes [28–30]. Table 1 summarizes characteristics of included studies.

**Table 1:** Summary of recent studies investigating the association between increased/Excessive Fetal Movements and perinatal outcomes

Author	Country	Year	Arms(*n*)	Design	Combinable outcomes	Method to identify fetal movement
Monari	Italy	2023	Adverse prenatal outcomes(*n* = 77) Control (*n* = 178)	Prospective case-control	IFM	Survey
Avraham	Israel	2023	Mothers with IFM (*n* = 153) Regular pregnancy (*n* = 299)	Prospective cohort	Delivery type-Maternal age- Meconium Aspiration-Birth weight- APGAR < 7- SGA-LGA-GDM-HTN-Labor induction	IFM sensation
Cohen	Israel	2022	Mothers with IFM (*n* = 282) Regular pregnancy (*n* = 43,432)	Retrospective cohort	Meconium aspiration- Umbilical cord around the neck- Birth weight- APGAR < 7- SGA-LGA-NICU Admission	IFM sensation
Sharp	UK	2021	Mothers with IFM (*n* = 64) Normal pregnancies (17,072)	Prospective cohort	Delivery type-Labor induction-NICU Admission	IFM sensation
Huang	China	2019	Mothers with IFM (*n* = 219) Healthy women who had undergone regular childbirth (278)	Prospective cohort	Delivery type-Maternal age- Umbilical cord around the neck- Birth weight-APGAR < 7-SGA-LGA-GDM-HTN-NICU Admission	Survey
Heazell	UK	2017	Stillbirth at, or after 28 weeks of gestation (*n* = 153) Regular pregnancy (*n* = 480)	Case-control	IFM	Online survey
Stacey	New Zealand	2011	Stillbirth at, or after 28 weeks of gestation (*n* = 155) Ongoing pregnancies(*n* = 310)	Case-control	IFM	Interview

IFM: Increased fetal movement. SGA: Small for gestational age, LGA: Large for gestational age, GDM: Gestational diabetes mellitus, HTN: Hypertension, NICU: Neonatal intensive care unit

### Risk of bias in studies

No significant bias was identified among included studies (Supplementary file).

### Cohort studies

#### Type of delivery

**Fig. 2:**
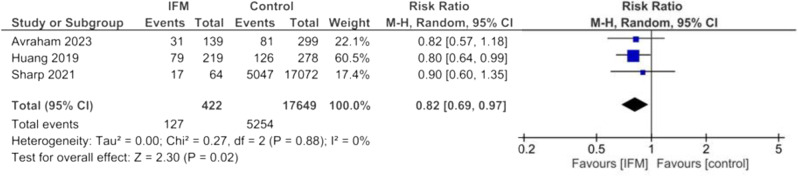
Forest plot for random-effect meta-analysis comparing risk of cesarean delivery between increased fetal movement patients and control group

Pooled risk ratio of cesarean delivery was calculated among 422 patients with IFM and 17,649 controls in three studies. Risk of cesarean delivery was significantly lower among patients with IFM. Overall estimates are the following: Random effect model, RR = 0.82, 95%CI; [0.69-0,97], *P* = 0.02, Fig. 2. No significant heterogeneity was found among included studies: I^2^ = 0%, *tau*^*2*^ = 0.00.

### Birth weight

**Fig. 3:**
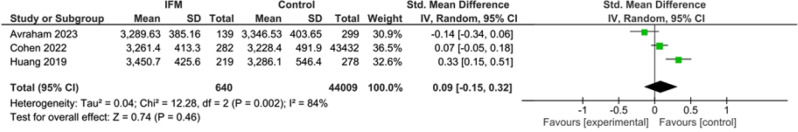
Forest plot for random-effect meta-analysis comparing standard birth weight mean difference between increased fetal movement patients and control group

Mean birth weights of 640 IFM patients and 44,009 controls were reported in three studies, pooled standard mean difference of birthweight was calculated via random effect model. Birth weight was not significantly different between two groups. Overall estimates are the following: SMD = 0.09, 95%CI; [-0.15, 0.32], *P* = 0.46, Fig. 3. There was high heterogeneity among findings of included studies. I^2^ = 84%, *tau*^*2*^ = 0.04.

### Small for gestational age

**Fig. 4:**
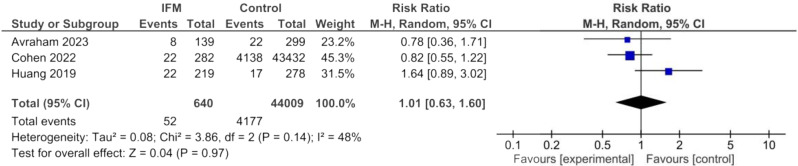
Forest plot for random-effect meta-analysis comparing risk of small for gestational age (SGA) between increased fetal movement patients and control group

Number of neonates small for their gestational age was reported in three studies including 640 IFM patients and 44,009 controls. Pooled risk ratio of SGA birth was not significantly different between two groups. Pooled estimates are the following: RR = 0.98, 95%CI [0.72, 1.33], *P* = 0.9, Fig. 4. No significant heterogeneity was found among studies: I^2^ = 0, *tau*^*2*^ *= 0.00.*

### Large for gestational age

**Fig. 5:**
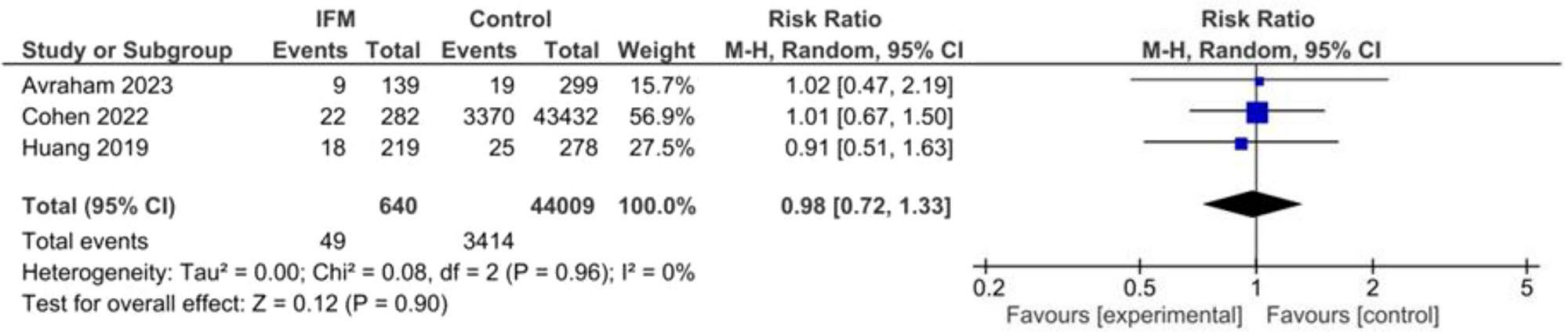
Forest plot for random-effect meta-analysis comparing risk of large for gestational age (LGA) between increased fetal movement patients and control group

LGA was reported in three studies including overall of 44,009 IFM cases and 640 controls. Similar to SGA, IFM was not significantly associated with higher risk of LGA birth. Summary estimates are the following: Random effect model, RR = 1.01, 95%CI; [0.63, 1.60], *P* = 0.97, Fig. 5. there was moderate heterogeneity among findings of these studies: I^2^ = 48%, *tau*^2^ = 0.08.

### NICU admission

**Fig. 6:**
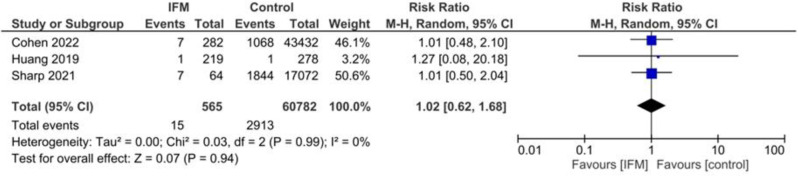
Forest plot for random-effect meta-analysis comparing risk of NICU admission between increased fetal movement patients and control group

Number of NICU admitted neonates were reported in three studies including 565 cases of IFM and 60,782 controls. Risk of NICU admission was not significantly different between two groups. Summary estimates are the following: Random effect model, RR = 1.02, 95%CI; [0.62, 1.68], *P* = 0.99, Fig. 6. No significant heterogeneity was present; I^2^ = 0, *tau*^*2*^ = 0.00.

### Maternal age

**Fig. 7:**
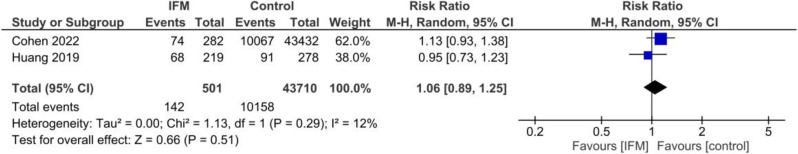
Forest plot for random-effect meta-analysis comparing maternal age between increased fetal movement patients and control group

Maternal age was reported in two studies, but Sharp et al. study only reported maternal age of IFM cases and did not report mean maternal age of controls. Standard mean difference of maternal age was not significantly different between two groups. Summary estimates are the following: SMD= -0.08, 95%CI; [-0.24-0.07], *P* = 0.28, Fig. 7.

### Umbilical cord around neck

**Fig. 8:**
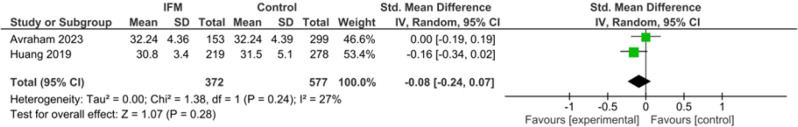
Forest plot for random-effect meta-analysis comparing rate of umbilical cord around neck between increased fetal movement patients and control group

Two studies including 502 IFM cases and 43,710 controls reported occurrence rate of umbilical cord around neck. Pooling via random effect model showed that IFM is not associated with higher or lower risk of umbilical cord around neck. Summary estimates are the following: RR = 1.06, 95%CI; [0.89, 1.25], *P* = 0.51, Fig. 8.

### GDM

**Fig. 9:**
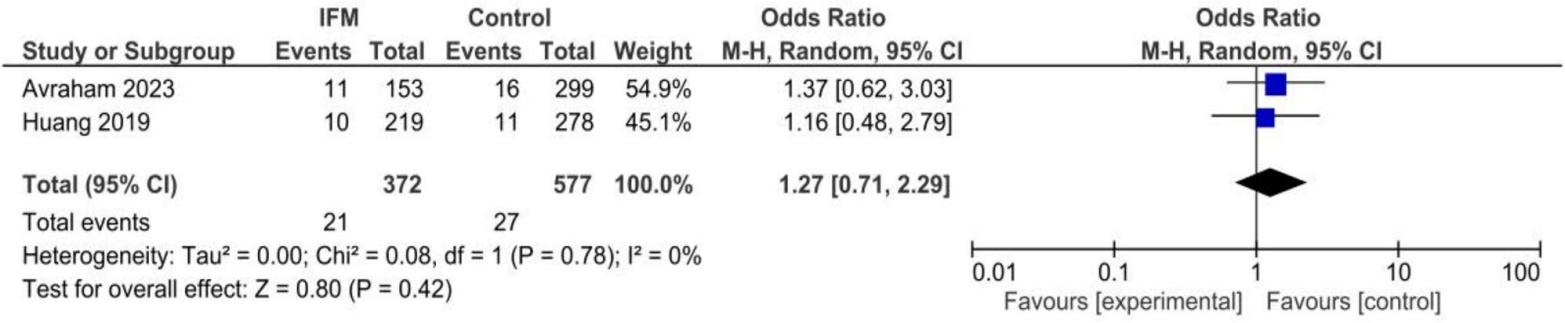
Forest plot for random-effect meta-analysis comparing prevalence of GDM between increased fetal movement patients and control group

Two studies including 372 cases of IFM and 577 controls reported GDM prevalence among included mothers. Pooling showed no significant association GDM and IFM: OR = 1.27, 95%CI; [0.71, 2.29], *P* = 0.78, I^2^ = 0, *tau*^*2*^ *= 0.00*, Fig. 9.

### HTN

**Fig. 10:**
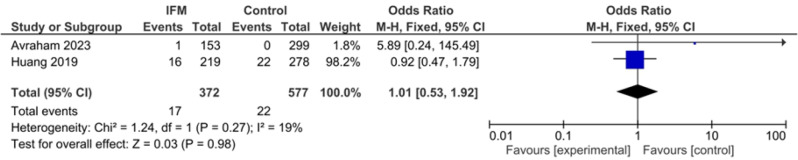
Forest plot for random-effect meta-analysis comparing prevalence of HTN between increased fetal movement patients and control group

Two studies including 372 cases of IFM and 577 controls reported HTN prevalence among included mothers. Pooling showed no significant association HTN and IFM: Fixed effect, OR = 1.01, 95%CI; [0.53, 1.92], *P* = 0.98, I^2^ = 0, *tau*^*2*^ *= 0.00*, Fig. 10.

### Induction

**Fig. 11:**
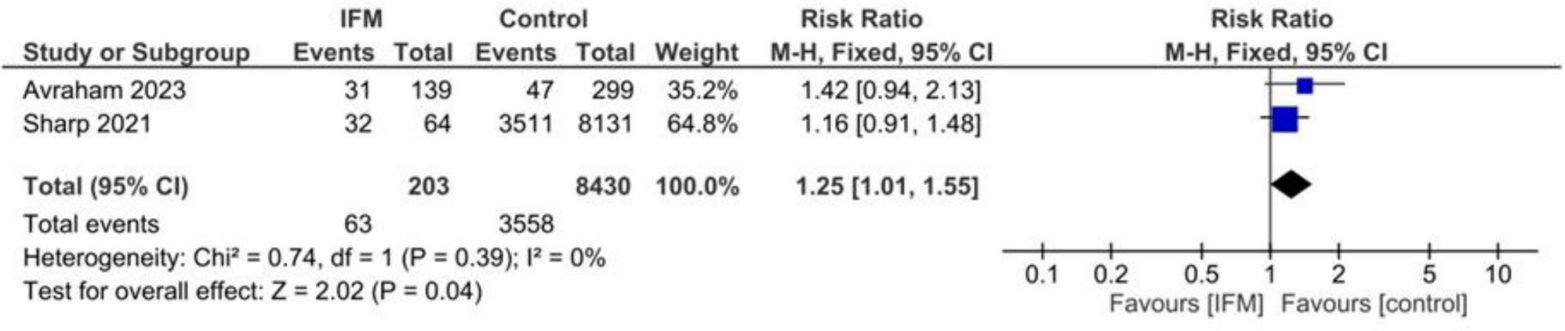
Forest plot for random-effect meta-analysis comparing number of cases underwenting labor induction between increased fetal movement patients and control group

Two studies including 203 cases of IFM and 8,430 controls reported number of cases underwenting labor induction. Pooling via fixed effect model showed that IFM is significantly associated with higher risk of labor induction; RR = 1.25, 95%CI; [1.01, 1.55], *P* = 0.04, I^2^ = 0, *tau*^*2*^ *= 0.00*,, Fig. 11.

### Case-control studies

#### Adverse perinatal outcomes

**Fig. 12:**
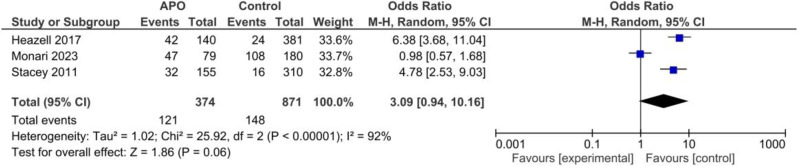
Forest plot for random-effect meta-analysis comparing odds of increased fetal movement between cases with adverse perinatal outcomes and regular pregnancy controls. Adverse perinatal events were defined as perinatal mortality (still birth and early neonatal mortality), operative delivery (cesarean section or vacuum) due to fetal distress, Apgar min 5 < 7, neonatal resuscitation at birth (including both invasive ventilation such as mechanical ventilation; non-invasive ventilation such as oxygen therapy, nasal CPAP, high flow), and NICU Admission

Three case-control studies including an overall of 374 cases with adverse neonatal outcomes and 871 controls reported IFM as one their outcomes. Pooled Odds of increased fetal movement was higher among cases compared to controls but it was not significant, random effect model, OR = 3.09, 95%CI; [0.94, 10.16], *P* = 0.06, Fig. 12.

## Discussion

The present study aimed to investigate the association between increased fetal movement (IFM) and various perinatal outcomes. To achieve this, a systematic review was conducted, resulting in the inclusion of 7 studies. The selected studies were analyzed to determine the relationship between IFM and outcomes such as type of delivery (cesarean delivery, induced or natural vaginal delivery), birth weight, small for gestational age (SGA), large for gestational age (LGA), neonatal intensive care unit (NICU) admission, maternal age, umbilical cord around the neck (UCNA), gestational diabetes mellitus (GDM), hypertension (HTN), and labor induction.

In terms of the type of delivery, the pooled analysis indicated that patients with IFM had a significantly lower risk of cesarean delivery compared to controls. It has been indicated that women with a gestational age of more than 37 weeks tend to experience a higher prevalence of IFM. Additionally, primigravid women have been found to have more episodes of excessive fetal movements compared to multiparous women [31]. This finding could potentially be attributed to a lower rate of cesarean sections in primigravid women and term pregnancies, or it could be an incidental observation.

However, when examining birth weight, no significant difference was observed between infants with IFM and controls. This implies that IFM does not seem to have a substantial impact on the birth weight of infants. Similarly, no significant difference in the risk of infants being born small for gestational age (SGA) or large for gestational age (LGA) was found between the IFM group and controls. These results suggest that IFM may not be a strong predictor of abnormal fetal growth. The analysis of NICU admission rates revealed no significant difference between neonates with IFM and controls. This suggests that IFM does not appear to be associated with an increased risk of neonatal complications requiring admission to the neonatal intensive care unit. Regarding maternal age, the pooled analysis did not find a significant difference between the IFM group and controls, indicating that increased fetal movement is not likely to be influenced by maternal age.

The occurrence rate of umbilical cord around the neck (UCNA) was also examined, and the results showed no significant association with IFM. This indicates that IFM is not a major factor contributing to the presence of UCNA during delivery. The prevalence of gestational diabetes mellitus (GDM) and hypertension (HTN) among mothers with IFM was also investigated. The analysis did not find a significant association between IFM and either GDM or HTN. However, it is worth noting that in the context of labor induction, the pooled analysis showed a significant association between IFM and a higher risk of undergoing labor induction. This finding implies that IFM may be a relevant factor in determining the need for labor induction.

In the case-control studies examining adverse perinatal outcomes including still birth, the pooled odds of increased fetal movement were higher among cases with adverse neonatal outcomes compared to controls. However, this result was not statistically significant, indicating that the relationship between IFM and adverse perinatal outcomes requires further investigation.

Consistent with the results of this study, a cohort study found a significant increase in vaginal delivery in the IFM group compared to the control group [31]. Similarly, another study reported a higher rate of induction of labor in women who experienced changes in fetal movement or reduced fetal movement compared to women with normal fetal movements [32].

In 1977, Sadowsky et al. [33], conducted a study that initially identified the sudden excessive movement of a fetus as a potential sign of acute fetal distress. Since then, numerous studies have been conducted to explore and better understand this phenomenon. Most of them suggested that increased fetal movements might be associated with Stillbirth or poor perinatal outcomes. As part of the STARS cohort, a web-based survey was used to study 1714 women who had experienced a singleton stillbirth at > 28 weeks gestation. Among them, 39% experienced unusual fetal movements, with 30.5% reporting significantly less movement and 8.5% reporting significantly more movement. In addition to the group that described only increased fetal movements, the number of women described increased movements before decreased movements and fetal deaths [21]. A similar finding was found in a Swedish study, where 10% of women described abnormally vigorous activity before stillbirth. A sudden increase in the movement was followed by limited or no movement and then fetal death [22].

Based on the Auckland Stillbirth Study, women who experienced a single episode of more vigorous movements had a seven-fold increased risk of stillbirth. Conversely, women who reported repeated episodes of the increased fetal movement were protected against stillbirth [28]. The findings of this study were replicated in the UK MiNES Study, in which women with a single episode of increased fetal movements had a two-fold risk of Stillbirth but a reduced risk if the episodes recurred [16, 29]. This team also demonstrated that women who reported increased strength of movements in the last 2 weeks had decreased risk of late stillbirth compared with those whose movements were not changed. Moreover, another study in New Zealand showed that maternal perceptions of more vigorous than usual fetal movements were associated with lower risks of late stillbirth [23].

Except for the increased or excessive fetal movements, fetal hiccups perception, duration, and frequency were also assessed to see the outcomes. Hiccup perception was assessed in 4 papers, reporting the negative effect of maternal perception of fetal hiccups on Stillbirth [16, 23, 28, 29].

Hazell et al. stated that in unadjusted analysis, daily hiccups or prolonged episodes of hiccups for more than 5 min can reduce stillbirth, while in adjusted analyses, it is not significant anymore. They also reported that the presence of hiccups did not make difference in the pregnancy outcomes between the case and control groups. Bradford et al. have demonstrated that increases in strength and frequency, and fetal hiccups are associated with a decrease in the incidence of stillbirth [13].

It was reported that women should expect the fetal movement to at least remain as strong or increase in late pregnancy. Women perceive these changes in strength differently, and some may not feel a stronger movement. The perception of the increased strength of movement may simply be due to the increased size of the fetus and the relatively limited space that makes movement more noticeable [23, 31].

Indeed, maternal perception of fetal hiccups is common and is associated with a reduced risk of late stillbirth. Fetal hiccups were first reported by Ferroni and are considered to be a normal part of fetal development [34, 35]. Increased maternal perception of fetal hiccups near term may result from greater fetal size, changes in fetal breathing, or neurological development. It may also result from increased recognition of fetal hiccups by the mother. Therefore, we can conclude that fetal hiccups are a normal part of pregnancy and are not associated with an increased risk of Stillbirth [5].

On the other hand, it is still unclear what underlying mechanisms lead to the excessive movements in the fetus; it may be caused by asphyxia, infection, an attempt to release cord entanglement, or a change in fetal behavior (causing signs of distress) in response to noxious stimuli [16]. Additionally, increased maternal anxiety may lead to an increased perception of fetal activity [36]. The evidence regarding excessive fetal movements is sparse; there is no clinical guidance regarding reporting this symptom and reducing the risk of subsequent Stillbirth. Cardiotocography and ultrasonography of the fetus and cord could be utilized at the presentation time to evaluate fetal seizures or umbilical cord entanglement [37].

It is possible to determine whether a mother has been exposed to an infection or noxious stimuli by examining her history and measuring the level of inflammatory markers or toxins in her blood [5, 38]. It is possible to assess maternal anxiety using validated anxiety scores [39]. In excessive fetal movements, fetal outcomes can be recorded after birth. Apgar scores, fetal acidaemia, or stress-related factors in umbilical cord blood can be used to diagnose perinatal asphyxia [40]. Placentas and cords can be systematically examined for signs of hypoxia, infection, or compression of the umbilical cord. Such studies would provide further evidence regarding the underlying cause of excessive fetal movement and how this symptom might relate to in-utero compromise and Stillbirth. As a result of this approach, we will be able to determine whether excessive fetal movements can be used alongside reduced fetal movements to reduce the risk of perinatal mortality.

The main strength of this study is an extensive collection of pregnancy-related variables from several countries and inclusion of studies with large number of participants. This study has some limitations; the main limitation is low number combinable studies although as mentioned in the methods section doing a meta-analysis is not impossible and included studies have large number patients, our study serves as a motivation for further research. case-control studies may be prone to recall bias due to their nature. However, these studies included interviewer-administered questionnaires, so they did not include hypotheses about the potential association of various patterns of movements that reduced this risk. Selection bias is also possible; however, the reasons for this would likely vary across countries, but the prevalence of fetal movement variables was relatively consistent. It is also noteworthy that we included different types of IFM assessment methods which could lead to bias.

## Conclusions

In conclusion, this systematic review and meta-analysis, which included seven studies, investigated the association between increased fetal movement (IFM) and various perinatal outcomes. The findings suggest that no statistically significant difference was found in birth weight, small or large for gestational age births, neonatal intensive care unit admission, maternal age, umbilical cord around the neck, gestational diabetes mellitus, or hypertension, implying that IFM may not be a major predictor of adverse perinatal outcomes or maternal conditions. Nevertheless, the significant association with increased labor induction warrants attention and further investigation. The study highlights the need for future research with larger sample sizes and standardized protocols to validate these associations and enhance our understanding of the impact of increased fetal movement on perinatal outcomes. Due to the limited number of studies included, the current findings should be interpreted with caution, and additional research is crucial to strengthen the evidence base in this area.

## Electronic supplementary material

Below is the link to the electronic supplementary material.

Supplementary Material 1

## Data Availability

The dataset supporting the conclusions of this article (i.e., data extracted from included studies) is available upon request to the corresponding author.
