# Supplementary material for: The association between increased fetal movements in the third trimester and perinatal outcomes; a systematic review and meta-analysis
Source: BMC Pregnancy Childbirth. 2024 May 15;24:365. doi: 10.1186/s12884-024-06547-3 (PMC11095027; doi:10.1186/s12884-024-06547-3)
Supplement: Supplementary file 1 — Supplementary Material 1 [file 12884_2024_6547_MOESM1_ESM.docx]

**Supplementary**

**Supplementary 1**

**Sensitive Search Strategy:**

**PubMed**

("Fetal movement*"[tiab] OR "fetal activit*"[tiab] OR "baby's movement*"[tiab] OR hypermotility*[tiab] OR "Baby movement*"[tiab] OR "Fetal Movement"[Mesh]) AND (outcome*[tiab] OR complication*[tiab] OR well-being[tiab] OR "well being"[tiab] OR distress[tiab] OR death*[tiab] OR surveillance*[tiab] OR "fetal health"[tiab] OR adverse[tiab] OR mortalit*[tiab] OR morbidit*[tiab] OR "Fetal Status"[tiab] OR stillbirth*[tiab] OR demise[tiab] OR compromise[tiab] OR Welfare[tiab] OR Mummification[tiab] OR Loss[tiab] OR Resorption[tiab] OR Fatalit*[tiab] OR "Pregnancy Outcome"[Mesh] OR"Fatal Outcome"[Mesh] OR "Stillbirth"[Mesh] OR "Fetal Death"[Mesh] OR "Mortality"[Mesh] OR "Morbidity"[Mesh] OR "Infant Welfare"[Mesh] OR "Obstetric Labor Complications"[Mesh] OR "Fetal Distress"[Mesh])

**Scopus**

TITLE-ABS-KEY(“Fetal movement*” OR “fetal activit*” OR “baby's movement*" OR hypermotility* OR "Baby movement*") AND TITLE-ABS-KEY(outcome* OR complication* OR well-being OR "well being" OR distress OR death* OR surveillance* OR "fetal health" OR adverse OR mortalit* OR morbidit* OR "Fetal Status" OR Stillbirth* OR demise OR compromise OR Welfare OR Mummification OR Loss OR resorption OR Fatalit*)

**ISI**

TS=("Fetal movement*" OR "fetal activit*" OR "baby's movement*" OR hypermotility* OR "Baby movement*") AND TS=( outcome*OR complication* OR well-being OR "well being" OR distress OR death* OR surveillance* OR "fetal health" OR adverse OR mortalit* OR morbidit* OR "Fetal Status" OR Stillbirth* OR demise OR compromise OR Welfare OR Mummification OR Loss OR Resorption OR Fatalit*)

**Embase**

("Fetal movement*" OR "fetal activit*" OR hypermotility* OR "Baby movement*"):ti,ab,kw AND (outcome* OR complication* OR well-being OR "well being" OR distress OR death* OR surveillance* OR "fetal health" OR adverse OR mortalit* OR morbidit* OR "Fetal Status" OR Stillbirth* OR demise OR compromise OR Welfare OR Mummification OR Loss OR Resorption OR Fatalit*):ti,ab,kw

**Supplementary 2**

**Critical appraisal tool**

**Case-control studies**

| Author | comparability | Group matching | Same inclusion criteria | Valid and standard Exposure measurements  For both groups | Same exposure measurement | Confounding factors identification | Presence of any Confounding factors dealing strategies | Valid and reliable outcome measurements | Meaningful exposure period length | Appropriate statistical analysis |
| --- | --- | --- | --- | --- | --- | --- | --- | --- | --- | --- |
| Heazell et al. | yes | yes | yes | yes | yes | yes | yes | yes | yes | yes |
| Stacey  et al. | yes | yes | yes | yes | yes | yes | yes | yes | yes | yes |
| Monari et al. | yes | yes | yes | yes | yes | yes | yes | yes | yes | yes |

**Cohort studies**

| Author | Groups similar population | Exposure measurements similar in both groups | Valid and reliable  measurements | Confounding factors identification | Presence of any Confounding factors dealing strategies | groups/participants free of the outcome at the start of the study | Valid and reliable outcome measurements | Meaningful exposure period length | Follow-up completion or reason of loss of follow-up explained | strategies to address incomplete follow up | Appropriate statistical analysis |
| --- | --- | --- | --- | --- | --- | --- | --- | --- | --- | --- | --- |
| Huang  et al. | yes | yes | yes | yes | yes | yes | yes | yes | yes | N/A | yes |
| Sharp  et al. | yes | yes | yes | unclear | unclear | yes | yes | yes | yes | N/A | yes |
| Avraham et al. | yes | yes | yes | yes | yes | Yes | Yes | yes | Yes | NA | Yes |
| Cohen et al. | yes | yes | yes | yes | Unclear | yes | yes | Yes | yes | NA | yes |
